# Validation of Two Commercial Multiplex Real-Time PCR Assays for Detection of SARS-CoV-2 in Stool Donors for Fecal Microbiota Transplantation

**DOI:** 10.3390/microorganisms10020284

**Published:** 2022-01-26

**Authors:** Vincenzo Di Pilato, Fabio Morecchiato, Cosmeri Rizzato, Gianluca Quaranta, Roberta Fais, Claudia Gandolfo, Alberto Antonelli, Maria Grazia Cusi, Mauro Pistello, Gian Maria Rossolini, Maurizio Sanguinetti, Antonella Lupetti, Luca Masucci

**Affiliations:** 1 Department of Surgical Sciences and Integrated Diagnostics (DISC), University of Genoa, 16132 Genoa, Italy; 2 Microbiology and Virology Unit, Florence Careggi University Hospital, 50134 Florence, Italy; alberto.antonelli@unifi.it (A.A.); gianmaria.rossolini@unifi.it (G.M.R.); 3 Department of Experimental and Clinical Medicine, University of Florence, 50134 Florence, Italy; fabio.morecchiato@unifi.it; 4 Department of Translational Research and of New Technologies in Medicine and Surgery, University of Pisa, 56127 Pisa, Italy; cosmeri.rizzato@unipi.it (C.R.); roberta.fais91@live.it (R.F.); mauro.pistello@unipi.it (M.P.); 5 Department of Laboratory Sciences and Infectious Diseases, Fondazione Policlinico Universitario A. Gemelli IRCCS, 00168 Rome, Italy; gquaranta88@gmail.com (G.Q.); maurizio.sanguinetti@policlinicogemelli.it (M.S.); 6 Department of Medical Biotechnologies, University of Siena, 53100 Siena, Italy; claudia.gandolfo@unisi.it (C.G.); mariagrazia.cusi@unisi.it (M.G.C.); 7 Microbiology, Fondazione Policlinico Universitario Agostino Gemelli IRCCS, Catholic University of Sacred Heart, 00168 Rome, Italy

**Keywords:** FMT, RT-PCR, COVID-19, feces, donor screening

## Abstract

Recurrent infection by *Clostridioides difficile* has recently been treated by fecal microbiota transplantation (FMT). As viable SARS-CoV-2 was recovered from stool of asymptomatic individuals, the FMT procedure could be a potential risk of SARS-CoV-2 transmission, thus underlying the need to reliably detect SARS-CoV-2 in stool. Here, we performed a multicentric study to explore performances of two commercially available assays for detection of SARS-CoV-2 RNA in stool of potential FMT donors. In three hospitals, 180 stool samples were spiked with serial 10-fold dilutions of a SARS-CoV-2 inactivated lysate to evaluate the Seegene Allplex™ SARS-CoV-2 (SC2) and SARS-CoV-2/FluA/FluB/RSV (SC2FABR) Assays for the detection of viral RNA in stool of FMT donors. The results revealed that both assays detected down to 2 TCID_50_/mL with comparable limit of detection values, SC2 showing more consistent target positivity rate than SC2FABR. Beyond high amplification efficiency, correlation between C_T_ values and log concentrations of inactivated viral lysates showed R^2^ values ranging from 0.88 to 0.90 and from 0.87 to 0.91 for the SC2 and SC2FABR assay, respectively. The present results demonstrate that both methods are highly reproducible, sensitive, and accurate for SARS-CoV-2 RNA detection in stool, suggesting a potential use in FMT-donor screening.

## 1. Introduction

Severe acute respiratory syndrome coronavirus 2 (SARS-CoV-2), the causative agent of the coronavirus disease-2019 (COVID-19), has caused a pandemic affecting the world population at a global scale and remains a major public health threat [1].

Even though people infected by SARS-CoV-2 exhibited a wide range of symptoms, COVID-19 is typically considered a respiratory disease, with primary manifestations including cough, sore throat, congestion, anosmia, and dyspnea. Nevertheless, gastrointestinal (GI) symptoms have been also recognized as manifestations of the disease [2,3,4]. Accordingly, even though the transmission of SARS-CoV-2 typically occurs through the respiratory tract, the recovery of viable viruses from stool of asymptomatic individuals and patients have been also reported (i.e., sometimes well after their respiratory infection has cleared), as well as abundant gastrointestinal glandular cell ACE-2 expression (the target receptor for SARS-CoV-2) and active replication within enterocytes [2,5,6,7,8]. Moreover, the detection of both viable SARS-CoV-2 and viral RNA in wastewater systems further underscore the potential role of the gastrointestinal tract in viral replication and shedding [9]. At present, however, a fecal–oral transmission route for SARS-CoV-2 remains to be definitively demonstrated.

In response to this issue, the U.S. Food and Drug Administration (FDA) released a safety alert about the risk of transmission of SARS-CoV-2 through fecal microbiota transplantation (FMT), a procedure that in recent years has clearly demonstrated its benefits in the management of recurrent *Clostridioides difficile* infection [10,11,12]. As such, FMT has been regarded as a non-postponable procedure to be continuously delivered during the COVID-19 pandemic, and specific recommendations have been released to reorganize the workflow of FMT during the pandemic to avoid the potential risk of transmission of SARS-CoV-2 through the fecal transfer and guarantee patient safety [13,14,15].

These recommendations included the expansion of donor screening with questionnaires and laboratory testing aimed at excluding SARS-CoV-2 infection, including detection of possible RNA traces in FMT donations [14]. However, development of stool tests has been slow, and an assessment of methods for detection of SAR-CoV-2 RNA in stool using commercial platforms has been provided in few cases [16,17], highlighting the need of reliable and robust methods are urgently needed.

The aim of this study was to validate and test performances of the Seegene Allplex™ SARS-CoV-2 and SARS-CoV-2/FluA/FluB/RSV Assays for the detection of viral RNA in stool of FMT donors.

## 2. Materials and Methods

### 2.1. Study Design

**Figure 1:**
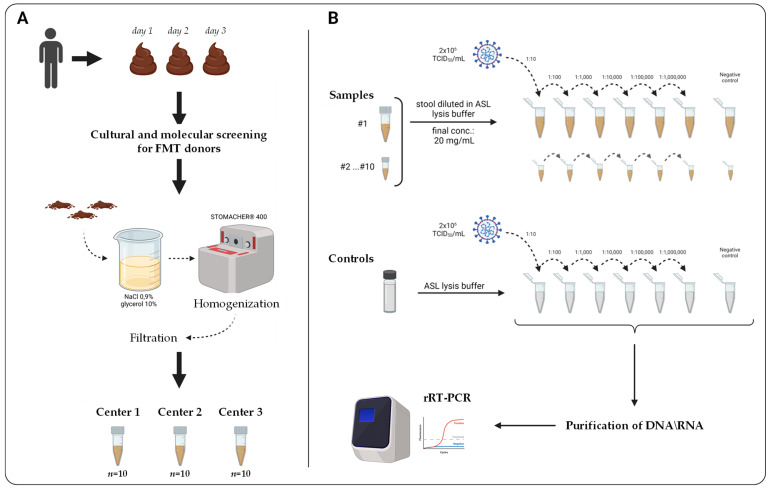
Overview of the study protocol used in this study. (**A**) Schematic representation of experimental phases performed by Center 1 concerning preparation of stool samples used in downstream analyses. (**B**) Schematic representation of experimental phases performed at each Center, including serial spiked dilutions of SARS-CoV-2 inactivated lysates and rRT-PCR testing.

This study involved three institutions in Italy, including the University Hospital “A. Gemelli” (Rome), the Florence Careggi University Hospital (Florence), and the Pisa University Hospital (Pisa)—hereinafter referred to as Center 1, 2, and 3—and consisted of two stages. Firstly, pilot experiments were carried out to establish a suitable quantity of stool that could be processed minimizing the effect of PCR inhibitors, that can be highly represented in this matrix, and can therefore affect the sensitivity and reproducibility of rRT-PCR assays [18]. To this purpose, stools from residual anonymized specimens from three SARS-CoV-2-negative subjects were collected at Center 2 and then pooled and diluted to a concentration of 50 mg/mL (the lowest concentration of starting material to be used with other Seegene Allplex™ diagnostic assays validated on stools) and 20 mg/mL. For each condition, seven aliquots were prepared and spiked with six serial 10-fold dilutions of SARS-CoV-2 inactivated lysates and subsequently tested using the SARS-CoV-2/FluA/FluB/RSV Assay, as described below. In a second stage, a multicentric evaluation was performed using pooled stool samples from a healthy subject recruited as potential FMT donor by Center 1. Overall, a total of 10 stool samples were available at each center for validating and testing two commercial multiplex real-time PCR assays for detection of SARS-CoV-2 in stool (Figure 1).

### 2.2. Stool Specimens and Donors’ Clinical Characteristics

Stool samples assayed in the multicentric evaluation phase of this study were obtained starting from three donations, on consecutive days in September 2021, from a healthy donor. Donor’s samples were delivered to the Microbiology laboratory of the Center 1, in accordance with the operational protocol, within six hours after evacuation to ensure anaerobic bacterial species preservation [19].

Donor selection is a very rigorous process. Firstly, the donor underwent a general questionnaire to exclude intestinal and extra-intestinal disorder. Subsequently, cultural and molecular analysis were performed on the donor’s stool to avoid the presence of any pathogen. Specifically, stool samples were analyzed in order to exclude the presence of *C. difficile* toxin A/B producer (LIAISON, DiaSorin Spa, Saluggia, VC, Italy) and intestinal pathogens, such as *Salmonella* spp., *Campylobacter* spp., *Shigella* spp., *Yersinia enterocolitica*, protozoa, and helminths. Moreover, the presence of vancomycin-resistant Enterococci (VRE), methicillin-resistant *Staphylococcus aureus* (MRSA) and gram-negative multi-drug resistant bacteria (MDR) was also excluded by cultural assay, using CARB/OXA chromID^®^ Agar, VRE Agar, SBL Agar and MRSA Agar (bioMérieux, Marcy-l’Etoile, France). Finally, RT-PCR Allplex™ Gastrointestinal Panel Assays (Seegene, Seoul, Korea) for the comprehensive detection and identification of common gastrointestinal pathogens (i.e., including the following bacteria: EAEC, EPEC, ETEC, STEC, *E. coli* O157, *Aeromonas* spp., *Campylobacter* spp., toxigenic *C. difficile*, *Salmonella* spp., *Shigella* spp./EIEC, *Vibrio* spp., and *Yersinia enterocolitica*; the following viruses: Adenovirus, Astrovirus, Norovirus GI\GII, Rotavirus, and Sapovirus; and the following parasites: *Blastocystis hominis*, *Cryptosporidium* spp., *Cyclospora cayetanensis*, *Dientamoeba fragilis*, *Entamoeba histolytica*, and *Giardia lamblia*) were performed in order to declare the stool “pathogens free” and then suitable for any procedure.

Once donation was elected as “suitable,” we proceeded with the preparation of the aliquots. Briefly, 60 g were suspended in 150 mL of sterile NaCl 0.9% and glycerol 10%, using a spatula. The homogenization and filtration phases were performed using a STOMACHER^®^ 400 Circulator (Seward Ltd., Worthing, UK). The program chosen was 260 rpm for 1 min. The stool suspension was then transferred from the bag into a sterile bottle and filtered with sterile gauzes to avoid the passage of any debris. Finally, 2 mL of the stool suspension were dispensed in sterile Eppendorf and stored at −80 °C.

### 2.3. Cell Culture and Propagation of SARS-CoV-2

Vero E6 cells were grown as a monolayer in Dulbecco’s modified Eagle’s medium (DMEM) (Euroclone, Milan, Italy) supplemented with 100 U/mL penicillin/streptomycin (Euroclone) and 5% heat-inactivated fetal calf serum (FCS) (Euroclone) at 37 °C in a humidified 5% CO_2_ atmosphere. SARS-CoV-2 wild-type (SARS-CoV-2/human/ITA/Siena-1/2020; GenBank: MT531537.2) and a B.1.1.7 (Alpha) lineage (GSAID EPI_ISL_1163688) strain were isolated from clinical swab and propagated on Vero cells until a cytopathic effect (CPE) appeared. Viral stocks were obtained and titrated on Vero cells. Both viral strains having a titer of 2 × 10^6^ TCID_50_/mL were heat-inactivated at 60 °C for 60 min [20], and stored at −80 °C until further use.

### 2.4. Preparation of Stool Samples Spiked with SARS-CoV-2 RNA

At each center, 10 stool samples were diluted in ASL Stool lysis buffer (Qiagen, Hilden, Germany) to a final concentration of 20 mg/mL by vortexing for at least 1 min, and seven aliquots were then prepared per each sample. Of these, six were spiked with serial 10-fold dilutions of SARS-CoV-2 (B.1.1.7)-inactivated lysates from 10^−1^ to 10^−6^ (i.e., equivalent to 2 × 10^5^–2 TCID_50_/mL), and one was used as negative control. Serial 10-fold dilutions of the viral-inactivated lysates were also prepared in ASL buffer alone to generate control standard curves (Figure 1).

### 2.5. RNA Extraction and rRT-PCR Testing

Stool samples were processed at once in Hamilton Microlab STARlet automated extraction and PCR setup system (Hamilton Company, Reno, NV, USA), using the STARMag 96 × 4 Viral DNA/RNA 200 C kit (Seegene Inc., Seoul, Korea).

For SARS-CoV-2 detection, two different commercial assays were used, namely Allplex™ SARS-CoV-2 (SC2; cat. no: RV10248X) and Allplex™ SARS-CoV-2/FluA/FluB/RSV (SC2FABR; cat. no: RV10259X) (Seegene Inc.). SC2 performs a multiplex reverse real-time PCR (rRT-PCR), being able to detect the following four viral targets: the envelope (E) gene, the RNA-dependent RNA polymerase (RdRP), the spike (S) gene, and the nucleocapsid (N) gene. An exogenous RNA-based internal control (IC) is also provided. The SC2FABR multiplex rRT-PCR is able to detect three viral targets (S, RdRP, and N) also with an exogenous RNA-based IC and an endogenous human-DNA-based IC. rRT-PCR were performed with a CFX96 thermal cycler (BioRad, Hercules, CA, USA), and results were interpreted with the SARS-CoV-2 Seegene Viewer Software v. V3.24.000 according to manufacturer’s instructions. The test results of each kit were interpreted following the manufacturer’s recommendations; viral targets detection was considered positive for a cycle threshold (C_T_) < 40.

For quantitative evaluation of SARS-CoV-2 viral load in control samples (i.e., diluted in ASL buffer), the Quanty COVID-19v2 (CLONIT Srl, Milan, Italy) PCR kit was used. rRT-PCR was performed with a CFX96 thermal cycler, and results were interpreted according to manufacturer’s instructions and normalized to obtain viral load expressed in both copies/mL and copies/mg stool.

### 2.6. Analytical Evaluations

Serial 10-fold dilution series of six concentration levels (i.e., from 2 × 10^5^ to 2 TCID_50_/mL) of inactivated viral lysates, with three measurements for each concentration, were used to evaluate: (i) a limit of detection (LoD), defined as the minimum concentration of nucleic acid that gave positive results for the RdRP and/or S and/or N targets in the majority (over 95%) of replicates tested [21]; (ii) accuracy, defined as the closeness in agreement between a single measurement and the true value of the analyte under investigation [22], and precision (repeatability), defined as the closeness of agreement between single test results on identical test material using the same method and aimed at measuring the random error (expressed as coefficient of variation, CV) of assays over a predetermined period of time by multiple measurements of aliquots derived from a homogeneous sample [21]; and (iii) linearity to assess the correlation between C_Ts_ and viral loads, expressed as amplification efficiency (ε) and coefficient of determination (R^2^) [21].

### 2.7. Statistical Analysis

Quantitative C_T_ comparisons were plotted and analyzed by linear regression analysis in GraphPad Prism (version 9.3.0). One-way ANOVA was performed to compare assays’ performances in the three centers and between samples and ASL. LoD was analyzed by logit regression analysis in SPSS Statistics software (version 28.0.1.0; IBM). *p*-Values of <0.05 were considered statistically significant.

## 3. Results

Here, we explored the performances of two commercially available assays that could be used to reliably detect the SARS-CoV-2 RNA in stool matrix. First, in order to evaluate a possible inhibitory effect exerted by an excessive stool matrix, which can negatively impact on rRT-PCR sensitivity and accurate pathogen detection, preliminary testing was performed using the SC2FABR assay (which additionally includes an endogenous amplification control compared to SC2) and spiked dilution of SARS-CoV-2 viral-inactivated lysates (ranging from 2 × 10^5^ to 2 TCID_50_/mL) into stool samples with a concentration of 50 mg/mL (the lowest concentration of starting material to be used according to manufacturer’s instructions) and 20 mg/mL. Results showed that the S\RdRP\N targets were detected in 11/18 (61%) and in 14/18 (77%) dilutions of stool samples concentrated at 50 and 20 mg/mL, respectively (Table S1). Of note, while both exogenous and endogenous controls were consistently detected over all tested dilutions, regardless of the matrix concentration, marked differences in C_T_ values were observed over all tested dilutions. Indeed, for all targets, lower C_T_ values were consistently observed in samples concentrated at 20 mg/mL compared to those concentrated at 50 mg/mL (Table S1). Taken together, these results suggest a decreased amount of starting stool material can markedly increase consistency of detection of SARS-CoV-2. Accordingly, all the following tests were carried out using the lowest stool concentration that gave reliable results (i.e., 20 mg/mL).

A multicenter comprehensive evaluation was then performed across three different hospitals (i.e., Center 1, 2, and 3), each processing 10 stool samples that were spiked with six 10-fold serial dilutions of SARS-CoV-2 viral-inactivated lysates (i.e., overall equivalent to 180 spiked samples), and rRT-PCR determinations were carried out using both commercial assays to estimate the variability, reproducibility, and consistency in detection of SARS-CoV-2 RNA (Figure 1).

Results of dilution experiments showed that both assays detected down to 2 TCID_50_/mL, which roughly corresponded to a technical LoD of 7 × 10^3^ copies of viral RNA per mg of stool matrix or to 1.33 × 10^2^ copies/µL, with mean C_T_ values at a 10^−6^-fold dilution ranging from 27.87 ± 2.26 to 30.11 ± 2.58 for the SC2 assay and from 27.52 ± 2.24 to 35.15 ± 1.95 for the SC2FABR assay. LoD values for the N target of SC2FABR, as per logit regression analysis, were 6.92 × 10^3^ copies/mg (CI: 2552–12349), corresponding to 13.84 × 10^3^ (CI: 5077–24720) copies/µL.

Although both assays had comparable LoD values, the SC2 was characterized by a more consistent target positivity rate (i.e., accuracy of 100%) than the SC2FABR, which exhibited slightly lower rates for the N target with dilution spiked with more than 2 × 10^2^ TCID_50_/mL (Table 1). Negative controls consistently showed expected results. Interestingly, in both assays, spiked dilutions with and without (i.e., ASL) stools showed a similar behavior (in terms of ΔC_T_) with the exogenous IC compared to SARS-CoV-2 target genes (Table 1 and Table S2).

**Figure 2:**
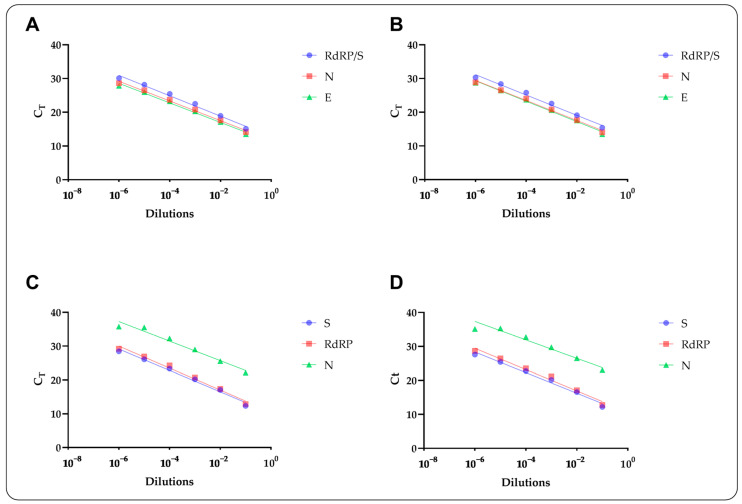
Global correlation between C_T_ values and log concentrations of inactivated viral lysates of SARS-CoV-2 observed for the SC2 (SARS-CoV-2) assay (**A**) and corresponding controls in ASL buffer (**B**) as well as the SC2FABR (SARS-CoV-2/FluA/FluB/RSV) assays (**C**) and corresponding controls in ASL buffer (**D**). Mean C_T_ values and standard deviation are shown per each dilution. rRT-PCR gene targets in each assay are identified by a color-based legend.

Assessment of linearity revealed a degree of correlation between C_T_ values and log concentrations of inactivated viral lysates, with R^2^ values ranging from 0.88 to 0.90 and from 0.87 to 0.91 for the SC2 and SC2FABR assay, respectively. Consistently, calculation of the PCR efficiency resulted in values greater than 108 and 114% for the SC2 and SC2FABR assay, respectively (Table 2, Figure 2). Endogenous control, on the other hand, displayed a linear dilution factor in half dilutions only (i.e., not detected in 10^−4^ to 10^−6^ dilutions), a behavior likely influenced by the host DNA present in cell cultures employed for spiked samples (Table 2).

Interlaboratory estimates of precision of both assays was overall comparable, with a total CV ranging from 4.78 to 12.29% for high-concentration samples (i.e., 2 × 10^5^ TCID_50_/mL) and from 5.55 to 8.91% for low-concentration samples (2 TCID_50_/mL) (Table 1). The CVs for exogenous and endogenous controls were in line with those of stool samples (Table S2). Analysis of variance (ANOVA) to compare assays’ performances between the three Centers revealed no statistically significant differences in variation of C_T_ per each dilution.

## 4. Discussion

Since the onset of the COVID-19 pandemic, the shedding of SARS-CoV-2 RNA in feces and the recognition of gastrointestinal symptoms in infected subjects have been documented by several studies [8,15,23,24,25], raising major concerns about a potential transmission of SARS-CoV-2 via the fecal–oral route [26,27,28]. However, to date, no studies have demonstrated a fecal matter-associated route of infection for SARS-CoV-2, and its transmission via stool specimens remains a topic of some debate, primarily concerning FMT treatment.

The U.S. Food and Drug Administration (FDA) has recently issued a safety alert about the risk of transmission of SARS-CoV-2 through FMT procedures [29], and additional recommendations have been released to avoid the potential risk of transmission of SARS-CoV-2 through fecal transfer and guarantee patient safety [26,27,28]. The potential fecal–oral asymptomatic transmission of SARS-CoV-2 has raised safety concerns for administering FMT globally; therefore, there is an urgent need for SARS-CoV-2 stool testing to be incorporated into FMT donor screening protocols in the COVID-19 era [13,30,31,32,33]. However, methods for the detection of the virus in stool have been poorly described.

Here, we present a technical evaluation of diagnostic performances of two IVD marked assays, namely the Seegene Allplex™ SARS-CoV-2 and SARS-CoV-2/FluA/FluB/RSV, for the detection of SARS-CoV-2 RNA in stool of potential FMT donors. Using accurate reference materials, including human processed stool from a healthy FMT donor and inactivated SARS-CoV-2 lysates, we have demonstrated sensitive and reproducible detection of SARS-CoV-2 RNA in stool. Present results also highlighted the need to accurately select the quantity of starting material, since it is well known that stool represents a highly heterogeneous sample matrix containing a plethora of PCR inhibitors (e.g., complex bile salts, urea, and glycolipids) that can negatively affect downstream molecular applications, including rRT-PCR [18]. In that regard, the use of endogenous amplification control may provide useful information to rule out the presence of potential PCR inhibitors or inefficient PCR conditions, indicating that dilution of the starting material may be required to obtain reliable results. Similarly, comparing the C_T_ value of the exogenous amplification control of stool sample and the one of ASL-negative control could be of help to confirm the absence of eventual inhibitors. None of the commercial assays to date validated for detection of SARS-CoV-2 RNA in stool adopted this approach [16,17].

A precise evaluation of the SARS-CoV-2 load in stools remains therefore technically challenging. Indeed, attempts to quantify the amount of SARS-CoV-2 RNA in stool samples yielded highly heterogeneous estimates ranging from 10^7^ to 10^2^ genome copies/mL [34]. According to the manufacturer’s specification, the SC2 and SC2FABR assays have a claimed LoD of 5000 RNA copies/mL and of 0.028 TCID_50_/mL, respectively, for nasopharyngeal swab specimens. Although in our experimental conditions a LoD as low as 133 RNA copies/µL (equivalent to 7 × 10^3^ copies of viral RNA/mg of stool matrix) has been determined, based on the high R^2^ and ε values, the technically assessed absence of PCR inhibitors (i.e., comparing C_T_ from ASL controls) and the log linearity behavior observed in serial dilution series of both assays, we would likely expect comparable performances in terms of LoD between respiratory specimens and the stool matrix. Among the few studies evaluating the LoD of SARS-CoV-2 RNA in stool using commercial platforms, one reported slightly higher LoD values for stool (i.e., 1250–2500 genome copies/mL) than upper respiratory specimens (i.e., 250–1000 copies/mL), a phenomenon most likely explained by the presence of PCR inhibitors in tested samples [16]; however, no specific evaluations have been performed to verify this hypothesis.

In terms of accuracy, SC2 and SC2FABR exhibited similar performances except for the N target, which showed a delayed gene positivity in dilution spiked with more than 2 × 10^2^ TCID_50_/mL in the latter assay. This peculiar pattern has been recently documented for the SC2FABR assay and was ascribed to a mutation occurring in the N nucleocapsid gene of SARS-CoV-2 belonging to the B.1.1.7 (Alpha) lineage [35].

Taken together, the present results demonstrate that both assays are highly reproducible, sensitive, and accurate for SARS-CoV-2 RNA detection in stool, with potential uses in FMT donor screening and in the release of quarantined FMT products.

This study, however, was beset with some limitations, including the lack of limiting conditions in dilution experiments to more accurately determine the LoD, and the need to evaluate the performance of the commercial assays here assessed using a larger number of stool samples. Additionally, although the SC2 and SC2FABR assays are routinely used for diagnosis of SARS-CoV-2 infections caused by B.1.1.529 (Omicron), B.1.1.617.2 (Delta) lineages from respiratory specimens, further experiments will be required to confirm their performance when assayed on the stool matrix.

## 5. Conclusions

Continued efforts in evaluating and validating molecular assays for detection of SARS-CoV-2 in stool are warranted to support adaptation of FMT donor screening and banking programs to the era of COVID-19 pandemic.

## Figures and Tables

**Table 1:** Overview of C_T_ values of rRT-PCR experiments performed with the Seegene Allplex™ SC2 (SARS-CoV-2) and SC2FABR (SARS-CoV-2/FluA/FluB/RSV) assays, using serial 10-fold spiked dilutions of SARS-CoV-2 viral-inactivated lysates in stool samples and controls (ASL). Quantitative evaluations of SARS-CoV-2 viral load have been also reported. Abbreviations: cp, copies; CV, coefficient of variation; SD, standard deviation; TCID, median tissue culture infectious dose.

SC2															
Dilution	Viral Titer (TCID_50_/mL)	RNA cp/µL	RNA cp/mg	RdRP/S (SD)	Percent Positivity (n/N)	CV (%)	C_T_^ASL^ (SD)	C_T_^N^ (SD)	Percent Positivity (n/N)	CV (%)	C_T_^ASL^ (SD)	C_T_^E^ (SD)	Percent Positivity (n/N)	CV (%)	C_T_^ASL^ (SD)
10^−1^	2 × 10^5^	1.03 × 10^7^	5.15 × 10^8^	15.12 (1.38)	100 (30/30)	9.13	15.42 (2.29)	14.10 (1.29)	100 (30/30)	9.15	14.04 (2.02)	13.51 (1.66)	100 (30/30)	12.29	13.49 (2.68)
10^−2^	2 × 10^4^	1.06 × 10^6^	5.30 × 10^7^	18.94 (1.59)	100 (30/30)	8.39	19.11 (2.83)	17.63 (1.27)	100 (30/30)	7.20	17.69 (1.96)	17.11 (1.57)	100 (30/30)	9.18	17.54 (2.33)
10^−3^	2 × 10^3^	9.71 × 10^4^	4.86 × 10^6^	22.51 (1.91)	100 (30/30)	8.49	22.57 (2.50)	20.80 (1.16)	100 (30/30)	5.58	20.82 (2.00)	20.28 (1.34)	100 (30/30)	6.61	20.62 (2.11)
10^−4^	2 × 10^2^	9.97 × 10^3^	4.99 × 10^5^	25.45 (1.44)	100 (30/30)	5.66	25.82 (2.33)	23.72 (1.15)	100 (30/30)	4.85	24.06 (1.59)	23.26 (1.28)	100 (30/30)	5.50	23.73 (1.60)
10^−5^	2 × 10^1^	9.27 × 10^2^	4.64 × 10^4^	28.18 (1.80)	100 (30/30)	6.39	28.37 (2.59)	26.53 (1.52)	100 (30/30)	5.73	26.54 (1.98)	25.99 (1.42)	100 (30/30)	5.46	26.45 (2.06)
10^−6^	2	1.33 × 10^2^	6.65 × 10^3^	30.11 (2.58)	100 (30/30)	8.57	30.33 (3.38)	28.61 (2.55)	100 (30/30)	8.91	28.89 (2.64)	27.87 (2.26)	100 (30/30)	8.11	28.73 (2.52)
**SC2FABR**															
**Dilution**	**Viral Titer (TCID_50_/mL)**	**RNA cp/µL**	**RNA** **cp/mg**	**RdRP/S** **(SD)**	**Percent Positivity** **(n/N)**	**CV (%)**	**C_T_^ASL^** **(SD)**	**C_T_^N^** **(SD)**	**Percent Positivity** **(n/N)**	**CV** **(%)**	**C_T_^ASL^** **(SD)**	**C_T_^S^** **(SD)**	**Percent Positivity** **(n/N)**	**CV (%)**	**C_T_^ASL^** **(SD)**
10^−1^	2 × 10^5^	1.03 × 10^7^	5.15 × 10^8^	12.75 (1.49)	100 (30/30)	11.69	12.88 (1.28)	23.01 (1.10)	100 (30/30)	4.78	22.16 (0.45)	12.17 (1.01)	100 (30/30)	8.30	12.34 (0.76)
10^−2^	2 × 10^4^	1.06 × 10^6^	5.30 × 10^7^	17.09 (1.20)	100 (30/30)	7.02	17.39 (0.63)	26.47 (1.09)	100 (30/30)	4.12	25.55 (0.62)	16.56 (0.93)	100 (30/30)	5.62	17.07 (0.57)
10^−3^	2 × 10^3^	9.71 × 10^4^	4.86 × 10^6^	20.68 (1.48)	100 (30/30)	7.16	20.74 (0.58)	29.76 (1.49)	100 (30/30)	5.01	29.02 (0.27)	19.79 (0.78)	100 (30/30)	3.94	20.20 (0.56)
10^−4^	2 × 10^2^	9.97 × 10^3^	4.99 × 10^5^	23.50 (0.93)	100 (30/30)	3.96	24.28 (0.56)	32.69 (1.43)	100 (30/30)	4.37	32.27 (0.99)	22.73 (0.86)	100 (30/30)	3.78	23.35 (0.88)
10^−5^	2 × 10^1^	9.27 × 10^2^	4.64 × 10^4^	26.40 (0.98)	100 (30/30)	3.71	26.90 (1.28)	35.21 (1.84)	80 (24/30)	5.23	35.55 (1.84)	25.40 (0.96)	100 (30/30)	3.78	26.08 (1.29)
10^−6^	2	1.33 × 10^2^	6.65 × 10^3^	28.55 (2.23)	100 (30/30)	7.81	29.20 (2.23)	35.15 (1.95)	50 (15/30)	5.55	35.77 (1.53)	27.52 (2.24)	100 (30/30)	8.14	28.44 (2.17)

**Table 2:** Evaluation of the linear dynamic range determined over six 10-fold serial dilution series for the SC2 (SARS-CoV-2) and SC2FABR (SARS-CoV-2/FluA/FluB/RSV) assays. Abbreviations: R^2^, coefficient of determination; ε, amplification efficiency.

		Stool Samples		ASL Controls	
Allplex™ Assay	Target	R^2^	ε (%)	R^2^	ε (%)
SC2	RdRP/S	0.88	114	0.84	115
	N	0.91	120	0.90	117
	E	0.90	121	0.88	114
SC2FABR	RdRP	0.91	114	0.96	107
	S	0.89	108	0.96	103
	N	0.87	133	0.94	122

## Data Availability

The data presented in this study are available on request from the corresponding authors.

## Supplementary Materials

The following supporting information can be downloaded at: https://www.mdpi.com/article/10.3390/microorganisms10020284/s1, Table S1: Correlation of C_T_ values and concentration of stool used in preliminary experiment performed with the Seegene Allplex™ SARS-CoV-2/FluA/FluB/RSV (SC2FABR) assays, spiking samples with serial 10-fold dilutions of SARS-CoV-2 viral-inactivated lysates. Quantitative (TCID_50_/mL) evaluations of SARS-CoV-2 viral load have been also reported. Abbreviations: TCID, median tissue culture infectious dose; ExoIC, exogenous rRT-PCR control; EndoIC, endogenous rRT-PCR control; nd: not detected; Table S2: Overview of CT values of control samples from rRT-PCR experiments performed with the Seegene Allplex™ SARS-CoV-2 (SC2) and SARS-CoV-2/FluA/FluB/RSV (SC2FABR) assays, using serial 10-fold spiked dilutions of SARS-CoV-2 viral-inactivated lysates in stool samples and controls (ASL). Quantitative evaluations of SARS-CoV-2 viral load have been also reported. Abbreviations: CV, coefficient of variation; SD, standard deviation; TCID, median tissue culture infectious dose; ExoIC, exogenous rRT-PCR control; EndoIC, endogenous rRT-PCR control.
