# Supplementary material for: Validation of Two Commercial Multiplex Real-Time PCR Assays for Detection of SARS-CoV-2 in Stool Donors for Fecal Microbiota Transplantation
Source: Microorganisms. 2022 Jan 26;10(2):284. doi: 10.3390/microorganisms10020284 (PMC8879890; doi:10.3390/microorganisms10020284)
Supplement: Supplementary file 1 [file microorganisms-10-00284-s001.zip › microorganisms-1547393-supplementary.pdf]

Table S1. Correlation of C_T values and concentration of stool used in preliminary experiments performed with the Seegene Allplex™ SARS-CoV-2/FluA/FluB/RSV (SC2FABR) assay, spiking samples with serial 10-fold dilutions of SARS-CoV-2 viral inactivated lysates. Quantitative evaluations of SARS-CoV-2 viral load ($TCID_{50}$/mL) have been also reported. Abbreviations: TCID, median tissue culture infectious dose; ExoIC, exogenous rRT-PCR control; EndoIC, endogenous rRT-PCR control; nd: not detected.

		Allplex™SARS-CoV-2/FluA/FluB/RSV Assay										
**Dilution**	**Viral titre** ($TCID_{50}$/mL)	**Stool conc.** (mg/mL)	**S**	**C_T**	**RdRP**	**C_T**	**N**	**C_T**	**EndoIC**	**C_T**	**ExoIC**	**C_T**
**10^{-1}**	$2x10^5$	50	+	21.90	+	21.88	+	27.12	+	28.28	+	30.64
**10^{-2}**	$2x10^4$		+	27.14	+	28.42	+	35.13	+	31.92	+	34.19
**10^{-3}**	$2x10^3$		+	29.66	+	30.11	+	36.18	+	36.24	+	32.06
**10^{-4}**	$2x10^2$		+	34.50	+	37.37	-	N/A	+	38.74	+	34.41
**10^{-5}**	$2x10^1$		-	N/A	-	N/A	-	N/A	+	38.38	+	34.99
**10^{-6}**	2		-	N/A	-	N/A	-	N/A	+	33.36	+	36.46
**10^{-1}**	$2x10^5$	20	+	19.05	+	19.53	+	22.06	+	30.25	+	28.91
**10^{-2}**	$2x10^4$		+	22.08	+	22.71	+	25.94	+	33.77	+	28.79
**10^{-3}**	$2x10^3$		+	25.91	+	28.01	+	32.47	+	36.79	+	30.29
**10^{-4}**	$2x10^2$		+	29.03	+	29.66	+	33.10	+	37.31	+	29.07
**10^{-5}**	$2x10^1$		+	33.14	+	34.14	+	35.03	-	*nd*	+	30.61
**10^{-6}**	2		-	N/A	-	N/A	-	N/A	+	37.40	+	31.07

Table S2. Overview of C_T values of control samples from rRT-PCR experiments performed with the Seegene Allplex™ SARS-CoV-2 (SC2) and SARS-CoV-2/FluA/FluB/RSV (SC2FABR) assays, using serial 10-fold spiked dilutions of SARS-CoV-2 viral inactivated lysates in stool samples and controls (ASL). Quantitative evaluations of SARS-CoV-2 viral load have been also reported. Abbreviations: cp, copies; CV, coefficient of variation; SD, standard deviation; TCID, median tissue culture infectious dose; ExoIC, exogenous rRT-PCR control; EndoIC, endogenous rRT-PCR control.

				Allplex™ SARS-CoV-2				Allplex™SARS-CoV-2/FluA/FluB/RSV Assays							
**Dilution**	**Viral titre** ($TCID_{50}$/mL)	**RNA cp/µL**	**RNA cp/mg**	**C_T^{ExoIC}** (SD)	**Percent positivity** (n/N)	**CV** (%)	**C_T^{ASL}** (SD)	**C_T^{EndoIC}** (SD)	**Percent positivity** (n/N)	**CV** (%)	**C_T^{ASL}** (SD)	**C_T^{ExoIC}** (SD)	**Percent positivity** (n/N)	**CV** (%)	**C_T^{ASL}** (SD)
**10^{-1}**	$2x10^5$	$1.03x10^7$	5.15E+08	23.53 (1.45)	100 (30/30)	6.16	23.34 (1.96)	27.21 (0.98)	100 (30/30)	3.60	27.59 (0.70)	23.28 (0.82)	100 (30/30)	3.52	22.13 (0.95)
**10^{-2}**	$2x10^4$	$1.06x10^6$	$5.30x10^7$	23.47 (1.58)	100 (30/30)	6.73	23.23 (2.01)	30.04 (0.99)	100 (30/30)	3.30	30.54 (0.64)	23.30 (0.97)	100 (30/30)	4.16	23.20 (0.76)
**10^{-3}**	$2x10^3$	$9.71x10^4$	$4.86x10^6$	23.68 (1.51)	100 (30/30)	6.38	23.89 (2.00)	32.98 (1.13)	100 (30/30)	3.43	32.96 (1.25)	23.42 (1.02)	100 (30/30)	4.36	23.75 (1.31)
**10^{-4}**	$2x10^2$	$9.97x10^3$	$4.99x10^5$	23.84 (1.31)	100 (30/30)	5.49	24.17 (1.76)	34.57 (1.43)	100 (30/30)	4.14	34.23 (1.97)	23.21 (0.91)	100 (30/30)	3.92	23.87 (1.32)
**10^{-5}**	$2x10^1$	$9.27x10^2$	$4.64x10^4$	23.98 (1.49)	100 (30/30)	6.21	24.17 (1.82)	34.44 (1.52)	83.33 (25/30)	4.41	35.69 (3.33)	23.12 (0.74)	100 (30/30)	3.20	23.62 (0.90)
**10^{-6}**	2	$1.33x10^2$	$6.65x10^3$	24.01 (1.56)	100 (30/30)	6.50	24.26 (2.08)	34.71 (1.63)	86.66 (26/30)	4.70	34.19 (-*)	23.31 (0.80)	100 (30/30)	3.43	23.47 (0.75)
